# Photonic Cavity Effects for Enhanced Efficiency in Layered Perovskite-Based Light-Emitting Diodes

**DOI:** 10.3390/nano11112947

**Published:** 2021-11-03

**Authors:** Lyuye Lin, Remo Proietti Zaccaria, Denis Garoli, Roman Krahne

**Affiliations:** 1 Istituto Italiano di Tecnologia (IIT), Via Morego, 30, 16163 Genova, Italy; lyuye.lin@iit.it (L.L.); denis.garoli@iit.it (D.G.); 2 Dipartimento di Chimica e Chimica Industriale, Universty of Genoa, Via Dodecaneso, 31, 16146 Genova, Italy; 3 CNITECH, Ningbo Institute of Materials Technology and Engineering, Chinese Academy of Sciences, 1219 Zhongguan West Road, Ningbo 315201, China

**Keywords:** light-emitting diodes, perovskite, external quantum efficiency

## Abstract

Layered architectures for light-emitting diodes (LEDs) are the standard approach for solution-processable materials such as metal-halide perovskites. Upon designing the composition and thicknesses of the layers forming the LED, the primary focus is typically on the optimization of charge injection and balance. However, this approach only considers the process until electrons and holes recombine to generate photons, while for achieving optimized LED performance, the generated light must also be efficiently outcoupled. Our work focuses on the latter aspect. We assume efficient photon generation and analyze the effects of the geometrical configuration together with the dipole orientation, mimicking the light emission, on the main characteristics defining the LED, such as the Purcell effect and the outcoupling efficiency. We find that in-plane dipoles result in significantly increased outcoupling efficiency. Furthermore, the mismatch in refractive index among the layers and their different thicknesses can be tuned to maximize the Purcell effect and minimize internal losses. The combined optimization of dipole orientation and layer thicknesses can improve the efficiency of the LED up to a factor 10, hence highlighting the importance of considering also the photonic properties of the LED structures if the objective is to maximize the LED performance.

## 1. Introduction

**Figure 1:**
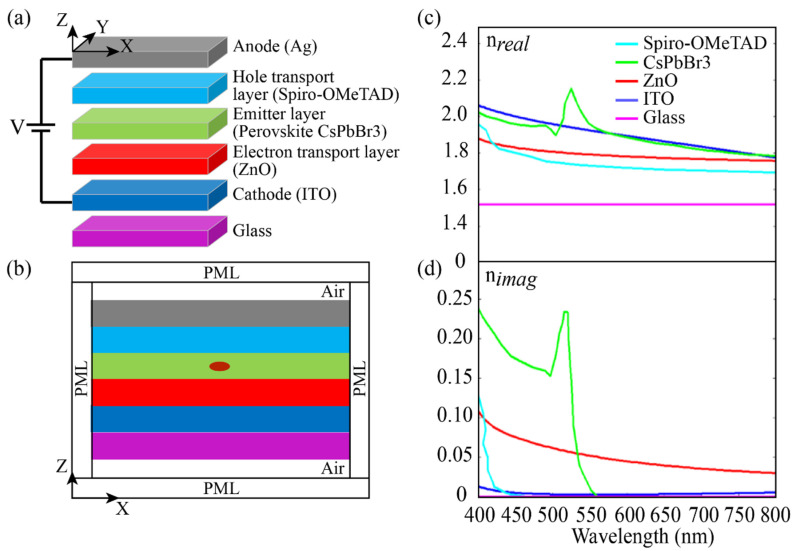
(**a**) General illustration of the device structure of the PeLED used for optical simulations. (**b**) Schematic illustration of the simulation model with boundary conditions and the Hertzian emitter (red dot) representing the photons emission. Real (**c**) and imaginary (**d**) of the refractive indexes of various materials in the PeLED.

Solution-processable nanomaterials with bright light emission, thus high photoluminescence quantum yield, are highly interesting for light-emitting diodes (LEDs), because they can be integrated in layered LED structures by low-cost deposition methods such as spin coating, doctor blading, etc. [1,2,3,4,5]. Colloidal dispersions of metal chalcogenide or metal-halide perovskite nanocrystals are among the most successful materials in this regard, and LEDs with external quantum efficiencies up to 23.4% have been reported [6]. The typical architecture of such LEDs consists of a planar transparent electrode coated with charge transport layers, the emitter layer, the complementary charge transport layers, and capped with an opaque counter electrode. Figure 1 depicts such an inverted structure consisting, from bottom to top, of a glass substrate, indium tin oxide (ITO) as the transparent cathode, zinc oxide (ZnO), CsPbBr_3_ film, the 2,2′,7,7′-Tetrakis [N, N-di(4-methoxyphenyl) amino]-9,9′-spirobi-fluorene (Spiro-OMeTAD), and silver (Ag) as the anode [1,4,5]. For our study, we take metal-halide perovskite light-emitting diodes (PeLEDs) as the demonstrator example, since perovskite nanocrystals have recently proven tremendous success in LEDs due to their high photoluminescence quantum yield, easy/wide bandgap tunability, high color purity, high brightness, and facile solution processability [5,7,8,9,10,11,12]. Through improvements on materials, morphology control, and structure optimization, PeLEDs achieved an external quantum efficiency ($\eta_{EQE}$) above 20% [11], which is impressive especially when considering that the external quantum efficiency of the first perovskite LED in 2014 was around 1% [7,13]. However, there are still large margins for further improvement, since most of the light emission from the perovskite films remains trapped in the LED structure and dissipated as heat. The main reasons are the mismatch of the refractive index between the emitter layer and both the ETL (electron transport layer) and HTL (hole transport layer) that causes multiple reflections within the LED, hence limiting the light outcoupling efficiency [8,9] as well as generating plasmonic coupling losses at the metallic anode. Other important aspects are the dependence of the external quantum efficiency on the dipole orientation of the emitters and the possible light enhancement by the Purcell effect that could be achieved by taking the different film thicknesses into account.

So far, most efforts have focused on analyzing and improving the optoelectronic properties of perovskite materials and charge transport layers in order to enhance the light extraction of perovskite LEDs [11]. However, few studies have addressed the structure of perovskite LEDs by studying their optical power loss [14]. In this respect, Shi et al. have quantitatively analyzed the effects of the thickness and refractive index of perovskite films on the PeLEDs light outcoupling efficiency [11]. Generally speaking, the external quantum efficiency, namely the ratio between the number of photons emitted from the LED to the number of injected charge carriers, is related to the internal quantum efficiency ($\eta_{IQE}$) and the light extraction efficiency (η, also known as outcoupling efficiency), according to the following expression:

(1) $$\;\eta_{EQE}=\eta \cdot \;\eta_{IQE}=\eta \cdot \gamma \cdot \eta_{S/T}\cdot q_{eff}.$$

Here, the outcoupling efficiency η is defined as the ratio between the number of photons emitted to free space from the LED to the number of photons generated in the emitter (perovskite) layer. The internal quantum yield $\;\eta_{IQE}$ is defined as the ratio between the number of photons generated in the emitter layer (not necessary all reaching the free space) to the number of injected charge carriers. Furthermore, $\eta_{IQE}$ can be decomposed into three factors, with γ describing the balance of the injected charges, $\eta_{S/T}$ representing the singlet/triple capture ratio at room temperature (for perovskite films, it can be taken equal to one [12,14]), and $q_{eff}$ representing the effective radiative quantum yield of the LED. In particular, γ can be tuned by selecting a suitable transport layer and by modifying its thickness [15], while $q_{eff}$ depends on defects and trapping of excitons [16] and can be related to the intrinsic quantum yield *q* (known also as photoluminescence quantum yield) of the perovskite film [15,17], representing the ratio between the photons generated within the emitter layer and the photon reabsorbed in the same layer:

(2) $$q_{eff}=\frac{q\cdot F}{q\cdot F+1-q}.$$

Here, *F* is the Purcell factor, which describes how the presence of a cavity (the perovskite layer) enhances the electron–hole recombination rate (i.e., the spontaneous emission) [11]. *F* can be calculated as:

(3) $$F=\frac{r_{pe}}{r_{0}}$$

where $r_{pe}$ and $r_{0}$ are the total radiative decay rates of a dipole emitter inside the PeLED structure and in free space, respectively [18]. Equations (1)–(3) show that $\eta_{EQE}$ mainly depends on the outcoupling efficiency η and on the Purcell factor *F*, therefore highlighting the need of a systematic and quantitative study especially of these two factors limiting the generation and emission of photons from a PeLED device. 

In this article, the optical power loss mechanisms of a layered PeLED device are methodically studied to improve $\eta_{EQE}$ by considering the role of the orientation of the emitting dipole (here representing the generated photon in the active layer) and by the Purcell factor *F* which, in turn, depends on the perovskite film thickness. 

## 2. Results and Discussion

The structure of the PeLED that we analyze as a demonstrator in this work is shown in Figure 1a. All the calculations are performed with the commercial software COMSOL Multiphysics, which is a finite element method-based software. From a geometrical point of view, the chosen structure is infinitely extended along the Y direction, while it is assumed to be as large as 8 μm along the X direction (see Figure 1b). This value, together with a minimum mesh size of 1 nm, was chosen to ensure the accuracy and reliability of the simulated results, as no change was observed for higher values at the selected wavelengths range. Furthermore, a Hertzian dipole (red dot) has been placed at the middle of the emitter layer to emulate a light-emitting source. Finally, a perfectly matched layer (PML) was assumed at the Z and X boundaries. 

The perovskite CsPbBr_3_ film (here defined by *q* = 0.9, *γ* = 1, $\eta_{S/T}=1$) [17,19,20] is sandwiched between ZnO (working as ETL) and Spiro-OMeTAD (working as HTL) [4,5]. All layers together form the photonic cavity associated to the LED. The real part of the refractive indexes (n*_real_*) of the materials composing the PeLED that we adopted in the present study are shown in Figure 1c [20,21,22,23,24]. Notably, there is a substantial difference in the refractive index values of ITO/perovskite versus the other materials. This mismatch is detrimental for the LED performance, as it leads to photon trapping within the LED structure due to total internal reflection (TIR) [8,9]. Figure 1d shows the corresponding imaginary part of the refractive index (n*_imag_*) that is strictly related to the power dissipated due to ohmic losses in the various layers. In particular, for CsPbBr3, a peak is found a little lower than 540 nm, suggesting the possibility for the occurrence of a sort of reabsorption effect (i.e., photons being generated and immediately absorbed [10]). Furthermore, CsPbBr3 is also known to possess a photoluminescence emission peak at 540 nm (direct bandgap ≈ 2.3 eV) [15], which is a number chosen as a reference value for the present study. Similarly, the Ag anode is implemented by following a Drude–Lorentz description [25,26,27].

As mentioned, the CsPbBr_3_ film is characterized by a complex refractive index that stems from its absorbance and emission properties. In particular, the reabsorption of emitted photons in the film (defined as reabsorption process) is possible and must be considered in our model in a way to match the actual behavior of perovskite films. In this respect, the value of the intrinsic quantum yield *q* which, as mentioned, takes into account the reabsorption phenomenon, had to be taken equal to 0.9 [16,19]. From a numerical point of view, this action was taken by adopting a sandwich-like structure for describing the emitting layer [28] that is formed by a loss-free (reabsorption-free, i.e., fully radiative central layer) material with electron–hole recombination layers around it, as shown in the inset of Figure S1. We quantify the specific dimensions of the sandwich-like emitter layer by using ρ to describe the ratio between the thickness d of the radiative layer and the thickness D of the full emitter layer: ρ = d/D. Figure S1 plots the intrinsic quantum yield *q* at 540 nm vs. D and ρ. As mentioned, for our further modeling, we set *q* = 0.9 by taking D = 30 nm and ρ = 0.6. In this work, we adopt an oscillating electrical dipole model to represent the light generated in the emitter layer (see Figure 1d). For an isotropic dipole orientation, the total radiated power and average Purcell factor both yield a linear relationship [12,13]:

(4) $$P_{total}=\frac{P_{⊥}+2*P_{∥}}{3}$$

(5) $$PF_{av}=\frac{PF_{⊥}+2*PF_{∥}}{3}.$$

**Figure 2:**
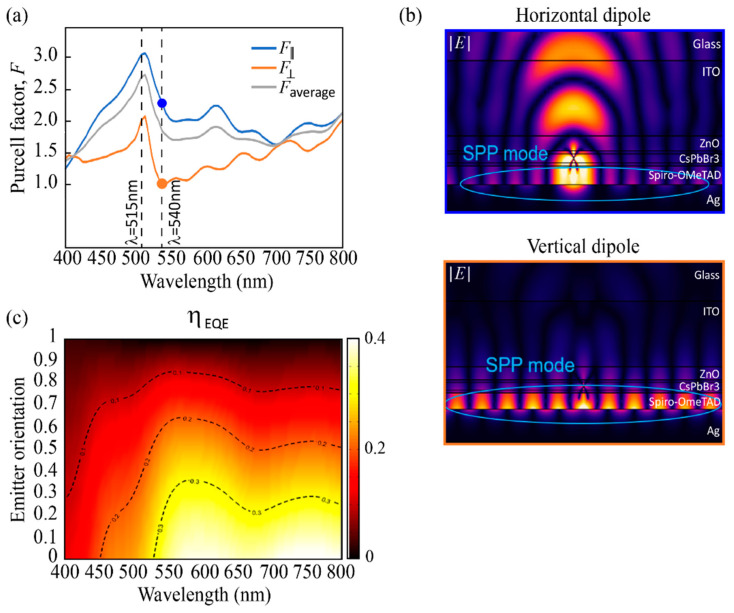
(**a**) Purcell factor for horizontal, vertical, and isotropic (average) dipole emitters as a function of wavelength. (**b**) The electric field plots describe the modulus of the electric field distribution in the PeLED in relation to the orientation of the emitting dipole and calculated at the wavelength of 540 nm (blue frame corresponding to the blue dot in (**a**). Similarly for the orange frame). (**c**) Profile of $\eta_{EQE}$ as a function of the emitting dipole orientation and wavelength. Emitter orientation equal to 0 means fully horizontal dipole. The y-axis describes the vertical dipole fraction (anisotropy factor equal to 0 means fully horizontal dipole).

Here, ⊥ and || represent the dipole orientation, which is either perpendicular (⊥, vertical) or parallel (||, horizontal) to the flat interface of the PeLED structure. Figure 2a shows the Purcell factor at different wavelengths for horizontal, vertical, and isotropic (average) dipole emitters located in the perovskite layer of a PeLED structure where the lateral dimensions of the layers are infinite, and their thicknesses were taken as 100 nm, 50 nm, 30 nm, 40 nm, and 200 nm for Ag, Spiro-OMeTAD, CsPbBr_3_, ZnO, and ITO, respectively. Furthermore, the glass substrate is considered semi-infinite along Z.

For an isotropic dipole distribution, the Purcell factor of the PeLED is 1.85 at the emission wavelength of 540 nm, and it shows a maximum of 2.77 at the 515 nm wavelength. Interestingly, the Purcell factor for a completely horizontal dipole distribution (2.31@540 nm, 3.11@515 nm) is much higher than for a vertical one (0.98@540 nm, 2.08@515 nm), which is caused by the electromagnetic emission pattern of an electric dipole, as evidenced in the electric field plots in Figure 2b and Supporting Information Figure S2. As the cavity Purcell factor affects the effective radiative quantum yield of the PeLED device, following Equation (1), a high concentration of horizontal dipoles results in enhanced $\eta_{EQE}$. The dependence of $\eta_{EQE}$ on the dipole orientation and emission wavelength is depicted in Figure 2c. For the isotropic case (corresponding to a dipole orientation of 0.33, see Equation (4), the maximum value for $\eta_{EQE}$ at 540 nm is 31.1%, while with completely horizontal dipole orientation, a $\eta_{EQE}$ of 37.58% can be obtained. For longer wavelengths such as 580 nm and 780 nm, the full horizontal dipole orientation can return a maximum $\eta_{EQE}$ value of 40%.

**Figure 3:**
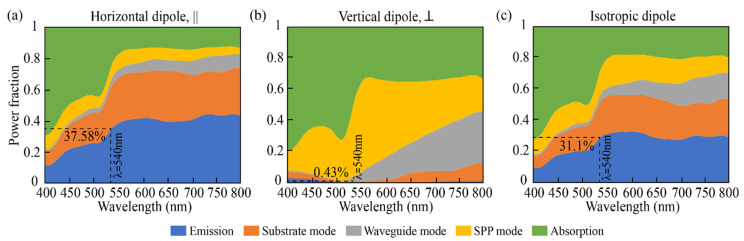
Reallocation of the perovskite emitted power in one of the five channels here considered: emission, substrate mode, waveguide mode, SPP mode, and absorption when horizontal (**a**), vertical (**b**), and isotropic dipole (**c**) are considered. The layers thicknesses are: 100 nm, 50 nm, 30 nm, 40 nm, and 200 nm for Ag, Spiro-OMeTAD, CsPbBr3 (with ρ= 0.6), ZnO, and ITO, respectively.

The emission of the perovskite layer can be channeled into air (PeLED emission), in localized optical modes (SPP mode at the interface Spiro-OMeTAD/Ag, as n*_real,perovskite_* > n*_real_,_Spiro-OMeTAD_*; substrate mode–mode sustained by the glass; waveguide mode–mode sustained by the different layers but glass) and be absorbed by the PeLED (non-radiative loss). This general description of the phenomena occurring in the PeLED is summarized in Figure 3, where emitting dipoles parallel, orthogonal, and isotropic to the surface of the perovskite layer are considered. At 540 nm and with fully horizontal dipoles (Figure 3a), nearly 38% of the optical power from the PeLED device appears as direct emission, therefore, still, a high portion is trapped in other channels. For vertical dipole orientation, the outcoupled emission is strongly reduced to 0.43% of the generated photons (Figure 3b), as most of the optical power generated from a vertical dipole couples into surface plasmon polaritons or waveguide modes, as seen in Figure 2b. The outcoupled radiative power for isotropic dipoles calculated by Equation (4) is instead 31.1%. Thus, the outcoupling efficiency can be improved by a factor of 1.2 by using emitters that are horizontally aligned with respect to an isotropic emitter layer [23]. This finding nicely correlates with reports on efficient LEDs with dot-in-rods as emitters that are typically organized in horizontal orientation [29,30]. 

**Figure 4:**
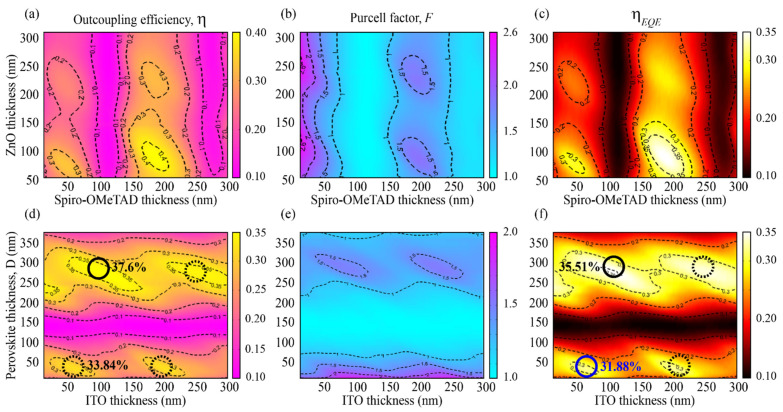
(**a**) Outcoupling efficiency η, (**b**) Purcell factor *F*, (**c**) $\eta_{EQE}$ of PeLED as functions of ZnO and Spiro-OMeTAD thicknesses at emission wavelength 540 nm (Ag = 100 nm, ITO = 200 nm, CsPbBr3 = 40 nm). (**d**) Outcoupling efficiency η, (**e**) Purcell factor *F*, (**f**) $\eta_{EQE}$ of PeLED as functions of ITO and perovskite thicknesses at emission wavelength 540 nm (Ag = 100 nm, Spiro-OMeTAD = 50 nm, ZnO = 40 nm). The circles represent local maxima. Isotropic dipoles distribution is assumed for all figures.

Next, we analyze the effects of the different layer thicknesses on the efficiency of the PeLED. We first address the two charge transport layers ETL (ZnO) and HTL (Spiro-OMeTAD), since their thickness is mainly determined by non-photonic aspects such as obtaining efficient and balanced charge injection. Figure 4a–c clearly show that the outcoupling efficiency η (Figure 4a), the Purcell factor *F* (Figure 4b), and $\eta_{EQE}$ (Figure 4c) can be optimized through the tuning of ZnO and Spiro-OMeTAD layers thicknesses. The results, obtained with fixed thicknesses of ITO and perovskite at 200 nm and 40 nm, respectively, indicate that they are sharply affected by the Spiro-OMeTAD thickness due to the interaction between the emitter dipole and the metal anode, whereas the effect of ZnO thickness fluctuates slightly. The reason is the location of the Spiro-OMeTAD layer, it being between the perovskite emitter and the metallic anode. This configuration represents an ideal cavity, in which the anode (Ag) is a good reflector in the visible range. For this reason, the Spiro-OMeTAD thickness plays an important role in the formation of constructive interference in the PeLED cavity. From Figure 4c, it is clear that there are four $\eta_{EQE}$ maxima, with two of them being the most important. The first one corresponds to $\eta_{EQE}$ equal to 31.9%, which is achieved for Spiro-OMeTAD and ZnO thicknesses of 50 nm and 40 nm, respectively. The second and most dominant peak is 40% when the thicknesses of Spiro-OMeTAD and ZnO are equal to 190 nm and 55 nm, respectively. However, it should be recalled that the thicker the ETL (or HTL), the longer the time required for the electrons to reach their destination. Thus, the thickness of ETL/HTL is required to be as thin as possible. Furthermore, Figure S3 describes the PeLED behavior when the perovskite thickness is changed from 40 to 90 nm with different ITO thicknesses, well confirming the ideal scenario provided by the 40 nm thickness. With these values fixed, we study the influence of the perovskite emitter layer and ITO layer thicknesses on the outcoupling efficiency, Purcell factor, and external quantum efficiency, as reported in Figure 4d–f.

In particular, Figure 4d displays the outcoupling efficiency η at λ = 540 nm considering an isotropic dipole distribution, where four local maxima can be noticed (highlighted by the continuous/dashed circles in the figure). These maxima are associated to waveguide modes forming inside the perovskite and ITO layers, and they show a stronger dependence on the perovskite layer thickness compared to ITO. Here, a change in the perovskite thickness can result in a significant increase in outcoupling efficiency up to a factor of 3.8 (from 0.1 of resonance to almost 0.38, full circle in Figure 4d). This increase mainly stems from the Purcell factor dependence on the layer thicknesses (see Figure 4e), thus on the performance of the LED as photonic cavity. The color map of the external quantum efficiency in Figure 4f manifests maxima in similar regions and consolidates that the performance of the LED can be significantly increased by optimizing the layer thicknesses, yielding over 35% efficiency for thick perovskite layers (thickness equal to 280 nm, ITO equal to 105 nm, full black circle in Figure 4f), 31.88% efficiency for thin perovskite layers (thickness equal to 40 nm, ITO equal to 65 nm, full blue circle in Figure 4f), and only 10% for the mismatched configuration. Thus, such layer optimization can influence the performance of the LED to a factor above 3. Interestingly, the thickness of the ITO layer can be freely adapted to obtain the highest possible external quantum efficiency, while the emitter layer thickness should be as small as possible to limit the light reabsorption process [31,32].

Another consideration resulting from Figure 4 is about the actual role played by the Purcell factor *F* in the definition of $\eta_{EQE}$. In fact, as shown by Equations (1) and (2), the quantity *F* influences $\eta_{EQE}$ through $q_{eff}$, which also depends on the quantity *q* ($\eta_{EQE}\propto q_{eff}\left(F,q\right)$); hence, any consideration regarding the behavior of $\eta_{EQE}$ should always take into account both *F* and *q*. In particular, it should be noticed that $\underset{F\to \infty }{\lim}q_{eff}=1$, regardless of *q*. In the present case, it was assumed that *q* = 0.9, with the maxima values obtained for *F* equal to 2.6 (Figure 4b) and 2.0 (Figure 4e). Interestingly, it can be observed that both these *F* values lead to very similar $q_{eff}$, around 0.95 (to be noticed that the maximum value for $q_{eff}$ is equal to 1); namely, *F* has a limited influence on $\eta_{EQE}$ with respect to *q*. It is indeed *q* that determines how fast $q_{eff}$ can approach its own limit 1; namely, the higher *q* is, the faster the $q_{eff}$ limit can be reached. In turn, this means also that high values of *q* determine a fast growth of $\eta_{EQE}$ upon an increase in the Purcell factor. Vice versa, for low *q* values, an increase in the Purcell factor would determine only a slight growth of $\eta_{EQE}$.

Finally, the effect of horizontal dipoles-only excitation on the overall LED performance should be highlighted. In this regard, Figure S4 demonstrates a maximum $\eta_{EQE}$ of 45.2% for a thick perovskite layer (280 nm), namely a 25% improvement with respect to the isotropic condition (Figure 4f), which is a situation occurring also for the thin perovskite layer (40 nm) with a maximum strong $\eta_{EQE}$ of 37.8% vs. 31.88% obtained for an isotropic dipole. Furthermore, it should also be highlighted the remarkable variation in $\eta_{EQE}$ upon geometry change, with a minimum around 4% corresponding to the off-resonance perovskite thickness (about 120 nm) and the aforementioned maximum of 45.2%, therefore an overall change close to 10 times. 

## 3. Conclusions

In summary, we performed a detailed analysis of the photonic properties of layered LEDs, taking a typical PeLED structure as the demonstrator. We identified and analyzed the main parameters characterizing the LED, namely the dipole orientation of the emitters, the outcoupling efficiency η, the Purcell factor *F*, the effective radiative quantum yield $q_{eff}$, and the external quantum efficiency $\eta_{EQE}$. Our results show that optimization of the photonic properties of LEDs can result in a significant improvement in performance, and we outlined a strategy of how this can be achieved by careful tuning of the thicknesses of the layers that constitute the LED. Considering that the material improvement is reaching its limits, with the photoluminescence quantum yield of the emitter layer close to unity and efficient and balanced charge injection by clever tailoring of the band structure of the LED, our work outlines complementary avenues to further improve the performance of the family of LEDs based on solution processable materials that are highly attractive for low-cost lighting devices.

## Data Availability

The data presented in this study are available on request from the corresponding author.

## Supplementary Materials

The following are available online at https://www.mdpi.com/article/10.3390/nano11112947/s1, Figure S1: Intrinsic quantum yield of emitter film, Figure S2: Effect of dipole orientation, Figure S3: Effect of perovskite thickness, Figure S4: All horizontal dipoles.
