# Supplementary material for: Photonic Cavity Effects for Enhanced Efficiency in Layered Perovskite-Based Light-Emitting Diodes
Source: Nanomaterials (Basel). 2021 Nov 3;11(11):2947. doi: 10.3390/nano11112947 (PMC8622141; doi:10.3390/nano11112947)
Supplement: Supplementary file 1 [file nanomaterials-11-02947-s001.zip › nanomaterials-1427749-supplementary.pdf]

Supporting Information for

Photonic Cavity Effects for Enhanced Efficiency in Layered Perovskite-Based Light-Emitting Diodes

Lyuye Lin [1,2], Remo Proietti Zaccaria [1,3,*], Denis Garoli [1] and Roman Krahne [1,*]

[1] Istituto Italiano di Tecnologia (IIT), Via Morego 30, 16163 Genova, Italy; lyuye.lin@iit.it (L.L.); denis.garoli@iit.it (D.G.)
[2] Dipartimento di Chimica e Chimica Industriale, Universty of Genoa, Via Dodecaneso, 31, 16146 Genova, Italy
[3] CNITECH, Ningbo Institute of Materials Technology and Engineering, Chinese Academy of Sciences, 1219 Zhongguan West Road, 315201 Ningbo, China
* Correspondence: remo.proietti@iit.it (R.P.Z.); roman.krahne@iit.it(R.K.)

Intrinsic quantum yield of emitter film

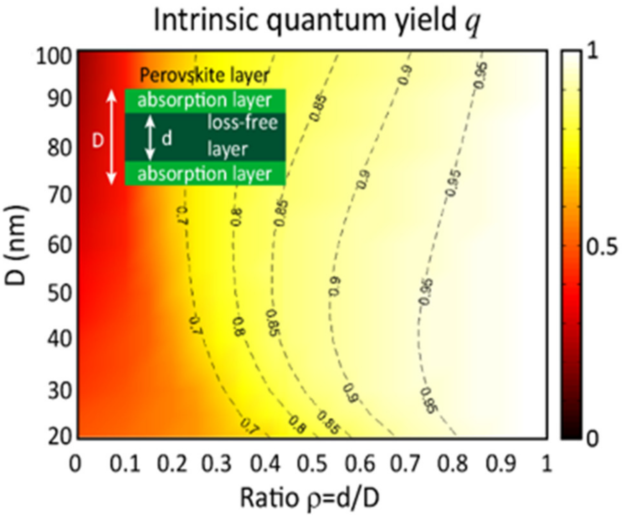

Figure S1. Intrinsic quantum yield at the absorption layer vs. the variation of the CsPbBr3 film thickness and the ratio ρ. The chosen wavelength is 540nm. The inset represents the schematic of the emitting layer.

Effect of dipole orientation

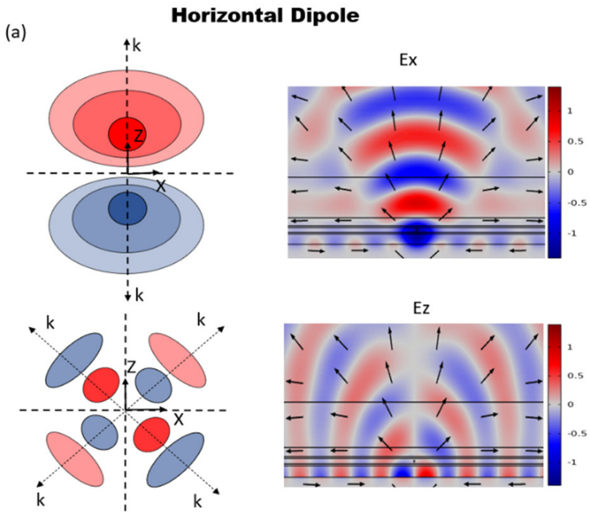

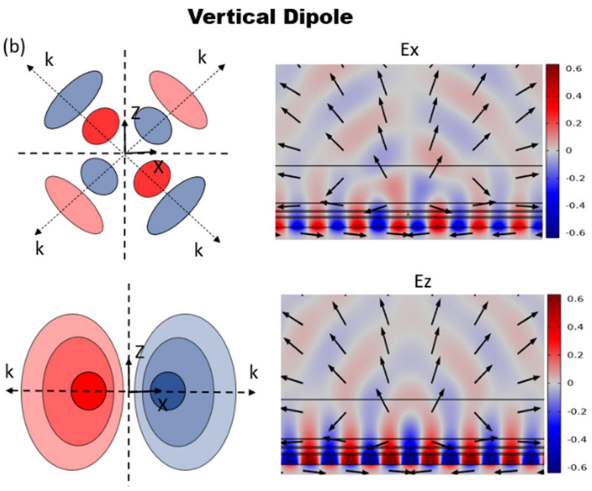

Figure S2. Distribution diagram of the electric field components (Ex、Ez) with a horizontal dipole (**a**) and vertical dipole (**b**) at resonance wavelength 540nm. The arrows represent the Poynting vector. The schemes show the PeLED (multilayer structure) surrounded (on the top) by air.

With a horizontal dipole, the associated electric charge oscillates along X-axis and the radiation energy mainly propagates along Z-direction (Figure S2(a)). The Ex-component dominates the radiation energy, showing also strong transferring of the electromagnetic radiation into air (outside the PeLED). For a vertical dipole, the electric charge oscillates along Z-axis and the radiated energy mainly propagates along X-direction. The Ez-component dominates the radiation energy. The electrical field is trapped at the interface of the Spiro-OMeTAD and Ag, and the radiation power is dissipated due to SPP loss. In addition, the electric filed intensity decreases along Z-axis, with a small transferring of the electromagnetic radiation to air (Figure S2(b)).

Effect of perovskite thickness

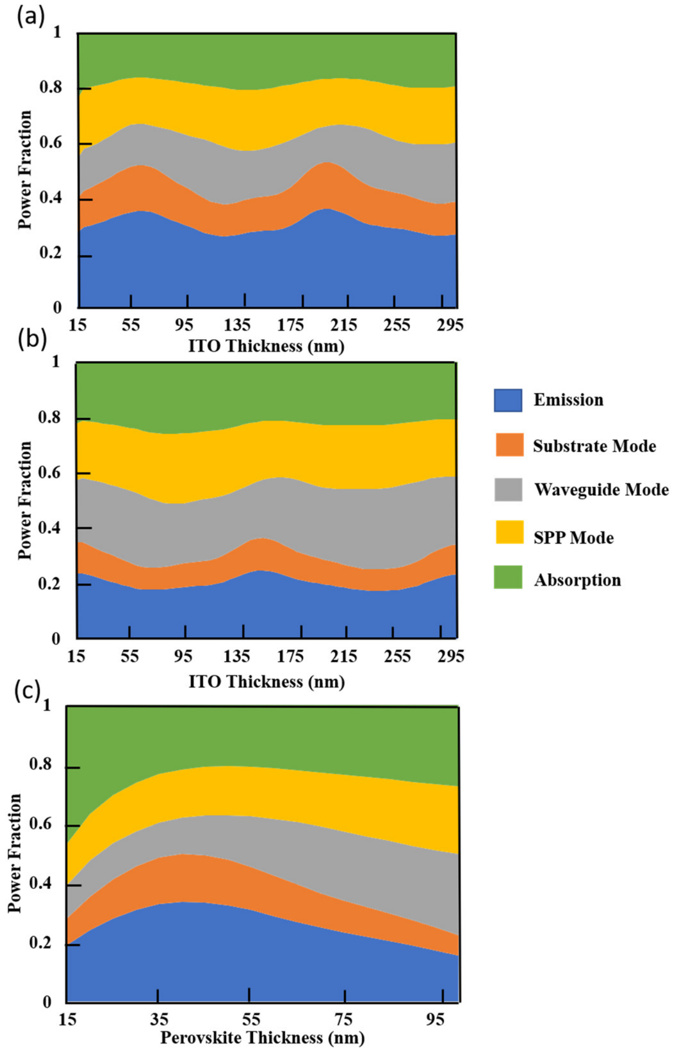

Figure S3. Power distribution in different optical modes of the PeLED with an isotropic dipole for various ITO thickness at emission wavelength 540nm. The thickness of the perovskite layer is fixed as 40nm (**a**) and 90nm (**b**). (**c**) Power distribution in different optical modes for various perovskite thicknesses at emission wavelength of 540nm. For all figures isotropic dipole configuration was considered with ITO thickness equal to 200nm and intrinsic quantum yield equal to 0.9.

Figures S3 reflects the amount of the optical power generated by the dipole coupled into different optical channels when perovskite CsPbBr3 thicknesses is equal to 40nm (corresponding to one of the maxima of Figure 4 main text) and 90nm are considered (taken as comparison term). For the 40nm perovskite CsPbBr3 film (Figure S3(a)), about 34.03% of optical power escapes to air, much higher than the 19.38% occurring in the case of 90nm perovskite CsPbBr3 film (Figure S3(b)). In particular, the 40nm perovskite CsPbBr3 case shows a 33.85% of the generated power coupled to air, with only 16.07% and 12.11% dissipated in the substrate and HTL-EML-ETL layers, respectively. Finally, 14.75% couples to SPPs and 23.04% is absorbed by the PeLED. When instead the 90nm perovskite CsPbBr3 is considered, more than 45% of the generated power is lost in waveguide and surface plasmon modes, while only 19.38% has the chance to couple into the air.

Figure S3(c) summarizes this situation showing the general PeLED behaviour upon change of the perovskite thickness. As the perovskite CsPbBr3 thickness increases from 15nm to 100nm, the emission to air of the PeLED raises to its maximum value (for perovskite film thickness equal to 40nm) and then gradually reduces.

All horizontal dipoles

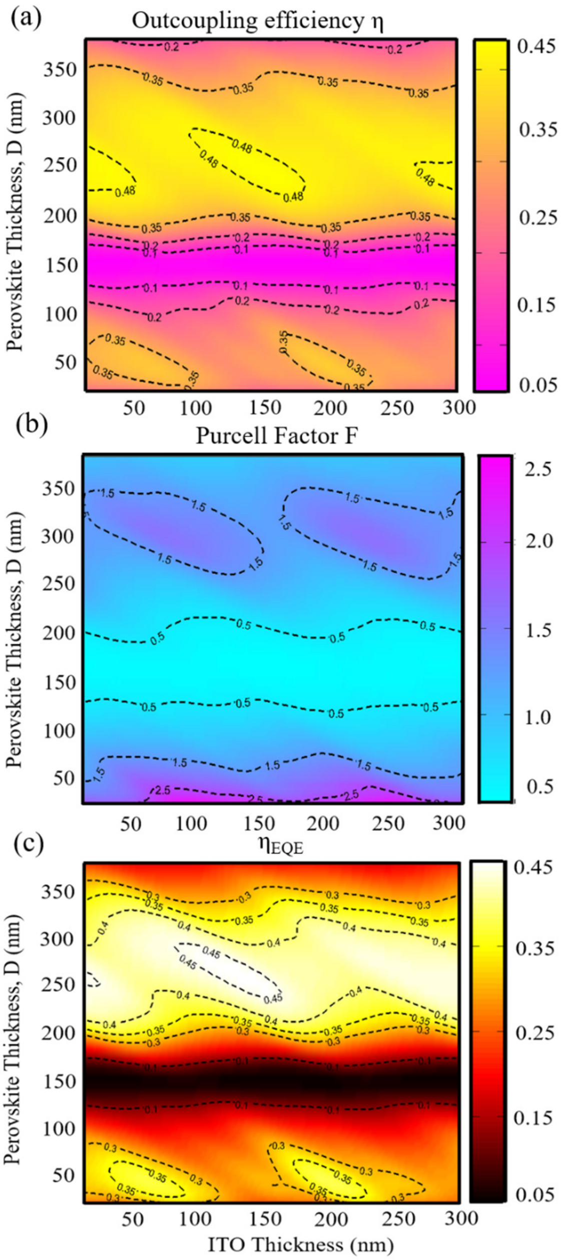

Figure S4. (**a**) Outcoupling efficiency η, (**b**) Purcell factor F, (**c**) η_{EQE} of PeLED as functions of ZnO and Spiro-OMeTAD thicknesses at emission wavelength 540nm (Ag=100nm, Spiro-OMeTAD=50nm, ZnO=40nm). For thin perovskite layer (40nm), the maximum for η_{EQE} is 37.8% (ITO layer equal to 200nm) while for thick perovskite layer (280nm) it is found to be equal to 45.2% (ITO layer equal to 110nm). For both situations the thicknesses of the remaining layers are 100nm, 50nm, 40nm for Ag, Spiro-OMeTAD and ZnO, respectively. The quantity ρ= 0.6 for all simulations. For all figures horizontal dipole configuration was considered.
